# Smart device interest, perceived usefulness, and preferences in rural Alabama seniors

**DOI:** 10.1038/s41597-024-03360-7

**Published:** 2024-05-22

**Authors:** Monica Anderson

**Affiliations:** The University of Alabama, Computer Science, Tuscaloosa, 35487 USA

**Keywords:** Scientific data, Information technology

## Abstract

Limited data exist on the preferences for smart home devices in rural Americans. We examined the interest, usefulness, and pleasantness of various smart home interfaces and determined whether they differed by ethnicity, rurality, age, and gender. A total of 118 older adults living in the rural Deep South completed a survey instrument that assessed interest in various smart home applications and was queried about the perceived usefulness and perceived ease of use of screen, voice, and robot interfaces in 7 distinct scenarios. Personality data was collected via the Big Five Inventory. Technology readiness was measured using a technological readiness instrument. Participants were primarily female (81%), rural (76%), African American (69%), and aged 65-74 years old (51%). Participants were recruited from a total of 5 cities in West Alabama within the Black Belt. Data was collected before COVID-19 (July 2018 through July 2019).

## Background & Summary

The Internet and Internet of Things (IoT) are redefining the function of the home much like electricity did in the previous century. Senior citizens and their caregivers have the most to benefit from this transition. New technologies can be leveraged to assist with tasks, monitor health status, assist caregivers, and ensure safety. Prior studies examining the attitudes of seniors regarding Internet and smart space technologies have relied primarily on samples from non-Hispanic white participants^1^. This is problematic because cultural factors may significantly impact the intention and adoption of technology. As a precursor to directed interventions in underserved populations, it is important to understand existing attitudes towards technology and ways that it can impact quality of life. This data could be used as a baseline in a similar population. In addition, this data can be used to determine similarities/dissimilarities between this and other populations.

This study takes place in the Black Belt, a region that stretches across Alabama and Mississippi and is known for its fertile land and a high percentage of African Americans. Although African Americans account for only 12-14% of the US population, they account for up to 40% of the population in the Black Belt with up to 30% living below the poverty level, which translates to $26,496 for a family of 4 (https://alabamapossible.org/programs/datasheet/). Other than Montgomery County (state capital), poverty rates for seniors 65 or older living in the Black Belt range from 8.7-23.1% with a state average of 10.2%. Even with lower life expectancies, African Americans 65 or older will increase from 9.2% to 15.9% of the population^2^.

The survey was administered pre-COVID (July 2018-2019). The administered survey is designed to assess the existing attitudes about the perceived usefulness of certain smart home devices individually or as part of routine household activities(adapted from^3^). Other scales include technological readiness^4^ and personality using the abbreviated Big Five Inventory 11-item survey^5^. Technological readiness data and/or personality may be correlated with preexisting perceptions about smart devices and user interface modalities.

### Key Models and Terminology

#### Unified Theory of Acceptance and the Use of Technology

Unified Theory of Acceptance and the Use of Technology questionnaire (UTAUT)^6^ identifies factors that may contribute or detract from sustained usage of technology. It suggests that acceptance of technology can be mediated by perceived usefulness and perceived ease of use, which drives sustained use of technology. Perceived usefulness describes the attitude that technology is useful in accomplishing a task. Perceived ease of use describes the attitude that technology is easy to use. Attitudes focus on beliefs, emotional responses, and behavioral tendencies regarding an object of thought. These attitudes may predict whether users will persist through a learning curve or subsequent disillusionment.

Additionally, the pleasantness of interfaces is collected from participants. Pleasantness is a subjective measure of a user’s positive emotional responses to a stimulus. In this case, the pleasantness rating focuses on a user’s understanding of a described technology feature. This measure captures the positive or negative attitude of the described technology based on how the user believes the technology would function.

#### Technology readiness

Technology readiness measures “people’s propensity to embrace and use new technologies for accomplishing goals in home life and at work"^7^. It is one of several aspects that impact the intent to use or adoption of technology.

#### Technological commitment

Technological commitment refers to attitudes that impact the willingness to use new or unfamiliar technology^4^. These attitudes focus on the user’s perception relative to their use of technology via three facets: technology acceptance, technology competence, technology control. It is predictive of other measures of technology acceptance.

#### Access and experience with technology and the Internet

Research dating back to the 1990s documents the gap in technology and Internet access for disadvantaged populations and its impact^8^. Many studies have linked access and experience with technology to attitudes related to technology. Cultural disconnect from technology could negatively influence adoption within a community. Disidentification (which can be described as the opposite of identification) occurs when people perceive behaviors as not being the norm in their social group^9^. Responses on individual and collective usage and experiences may correlate with certain attitudes about technology.

#### Big Five Personality Model

The Big Five Personality Model defines personality along five dimensions (neuroticism, extraversion, agreeableness, and conscientiousness and Openness). Neuroticism describes one’s confidence and emotional stability. Extraversion concerns whether a person draws energy being alone or interacting with others. Agreeableness concerns how well someone gets along with others. Conscientiousness concerns one’s ability to manage impulses and be goal-directed. Openness refers to one’s openness to experiences. It is a psycholexical approach to quantifying personality that relies upon a shared understanding of personality dimension terms^10^. Although there are competing personality models, there is evidence of the universality of the Big Five Personality Model^11^.

## Methods

### Study population

We recruited study participants from five Senior Activity Centers in the rural Deep South between July 2018 and July 2019. The Senior Activity Centers are central locations that provide socialization, education, and recreational activities for seniors living in designated areas. Each center also provides daily meals for those who attend the centers daily as well as home-delivered meals for those seniors in the community who have health limitations. Older adults interested in participating in a survey provided informed consent and completed a battery of survey questionnaires via paper. Upon completion of the surveys, older adults received a $5 gift card as an incentive for participation. A total of four rural sites and one urban site were recruited to participate in this study. The University of Alabama Institutional Review Board approved all procedures before the survey administration (17-11-725, 22-10-6007). All participants provided informed consent according to the approved protocol.

### Measures

To facilitate comparison and analysis, the survey was compiled from existing surveys and instruments. The survey collected perceptions on smart home technology along with personality and technological readiness as possible contributing factors. Demographic information was also collected.

#### Perceived usefulness of smart devices

Participants were asked to evaluate the perceived usefulness and perceived ease of use of four smart home applications: lights, fans, energy monitors (i.e., energy consumption monitors), and door locks (Table 1). Participants were asked to rate the smart devices about interest and automation. Interest-related questions asked: (a) to what extent were they interested in the technology and (b) how useful would the technology be if adopted? Automation-related questions asked about comfort with smart control of devices. All survey items were rated on a five-point Likert-type response scale ranging from 1 (Strongly Disagree) to 5 (Strongly Agree). The question on energy monitoring is interesting since many in this population seek financial assistance on paying electricity bills. There may be a financial incentive to use devices that regulate or reduce the cost of energy).

**Table 1:** Participants were asked to rate their attitudes regarding four smart home devices.

*These questions ask about your feelings about remotes that turn your lights off and on*.
Q3. I am interested in turning my lights on and off using a remote.
Q4. Turning my lights on and off using a remote would be useful.
Q5. I would be comfortable turning the lights and lamps on and off using a remote.
Q6. It is important that I have control when the remote can be used.
Q7. I want the system to learn when I want lights turned off and on.
*These questions regard your feelings about adjusting your fans and heat using a remote*
Q9. I would be interested in using a remote to adjust my fans and heaters
Q10. Using a remote to adjust my fans and heaters would be useful
Q11. I would be comfortable using a remote to adjust my fans and heaters.
Q12. It is important that I control when the remote can be used to adjust the fans and heaters
Q13. I want the computer to learn how I want the fans and heaters used
*These questions regard your feelings about receiving information about your electricity usage*.
Q15. I would be interested in seeing how much electricity my home is using
electricity usage.
Q16. Seeing how much electricity my home is using would be useful.
electricity usage.
Q17. I am comfortable knowing how much electricity my home.
electricity usage.
Q18. It is important that I control when energy information is collected
*These questions regard your feelings about remotes that can unlock the doors*.
electricity usage.
Q19. I would be interested in using a remote to unlock my doors.
Q21. Using a remote to unlock my door would be useful
Q22. I am comfortable using a remote to unlock my door makes me comfortable.
Q23. It is important that I control when the remote can unlock the door
Q24. It would be safe to use a remote to unlock doors

Participants answered using a 5-point Likert scale (Strongly Agree, Agree, Neither Agree or Disagree, Disagree, Strongly Disagree).

The technology acceptance model (TAM)^12^ describes how technology adoption is driven by perceived usefulness and perceived ease of use. Perceived usefulness describes the user’s attitude that the technology will assist the user with achieving a task, whereas perceived ease of use is the degree to which a person believes that using a particular system would be free from effort^12^. Although TAM is an acceptable model, additional factors must be used to understand and predict the long-term use of technologies in older adults^13,14^.

#### Pleasantness of interfaces

Participants were asked to respond to a series of scenarios in Table 2 used in Schiffhauer *et al*.^3^. Participants were asked How pleasant would it be to interact via (a) voice or speech recognition, (b) screen control, (c) robot, and (d) conventional. Participants responded to each scenario and smart application (e.g., screen control) on a 5-point Likert-type response scale ranging from 1(Very Pleasant) to 5 (Very Unpleasant).

**Table 2:** Scenarios based on work in Schiffhauer *et al*.^3^.

Scenarios	
Phone	Q25. The phone rings while I am washing the dishes. The person calling me is displayed on a screen above the kitchen sink. How pleasant would it be to interact via: 25.1) voice or speech recognition, 25.2) screen control, 25.3) robot or 25.4) conventional?
Doorbell	Q26. I am waiting for visitors and just as I am in the bathroom the doorbell is ringing. The person standing right in front of the door is displayed on a screen in the bathroom. How pleasant would it be to interact via: 26.1) voice or speech recognition, 26.2) screen control, 26.3) robot or 26.4) conventional?
TV	Q27. I am sitting on the couch while watching TV. I want to switch the channel, but the remote control is not available. How pleasant would it be to interact via: 27.1) voice or speech recognition, 27.2) screen control, 27.3) robot or 27.4) conventional?
Music	Q28. I am standing in the shower while listening to music. I would like to adjust the volume/change the title. How pleasant would it be to interact via: 28.1) voice or speech recognition, 28.2) screen control, 28.3) robot or 28.4) conventional?
Door	Q29. I return from a day of shopping loaded with bags and need to unlock the door. How pleasant would it be to interact via: 29.1) voice or speech recognition, 29.2) screen control, 29.3) robot or 29.4) conventional?
Email	Q30. While I am cleaning the kitchen, it occurs to me that I will also need to write an important e-mail. How pleasant would it be to interact via: 30.1) voice or speech recognition, 30.2) screen control, 30.3) robot or 30.4) conventional?
Light	Q31. I am having visitors and we are sitting in the kitchen while playing a parlor game. The light is slightly too dark and I want it to be a bit brighter so that we have a better view of the playing field. How pleasant would it be to interact via: 31.1) voice or speech recognition, 31.2) screen control, 31.3) robot or 31.4) conventional?

Each scenario asked a four-part question regarding *How pleasant would it be to interact via:* 1) Voice or speech recognition, 2) screen control, 3) robot, and 4) Conventional. Participants answered using a 5-point Likert scale (Very pleasant, Pleasant, Neither, Pleasant nor unpleasant, Unpleasant and Very unpleasant).

#### Technological commitment

 The technological readiness scale^4^ is a validated instrument (Table 3) designed to predict adaptability technology for senior citizens. Neyer *et al*. studied 825 participants and correlated related constructs in technology use, personality, successful aging, and health to determine construct validity. Technology readiness was measured using three subscales: acceptance, competence, and control beliefs. Acceptance is interest in technological innovation. Competence encapsulates previous experiences with technology and adaptation to future technology. Control beliefs measure the perceived influence and control of technology in a specific situations and their applicability in one’s life.

**Table 3:** Questions from technological readiness survey^4^.

Q32_1	I am very curious about new developments in computers and/or devices.
Q32_2	For me, dealing with computers and/or devices is usually too much work.
Q32_3	I find dealing with new computers and/or devices difficult - I usually cannot do it.
Q32_4	It is in my hands whether I can succeed in the use of computers and/or devices - with chance or luck, this has little to do.
Q32_5	I am always interested in using the latest computers and/or devices
Q32_6	I am often afraid of dealing with modern computers and/or devices.
Q32_7	If I have difficulties with the handling of computers and/or devices, it depends ultimately only on me that I solve it.
Q32_8	If I had the opportunity to do so, I would use much more computers and/or devices than I do.
Q32_9	I am afraid that new computers and devices are more likely to be broken than I am using them properly.
Q32_10	What happens, when I deal with computers and new devices, is ultimately my control.
Q32_11	I quickly find the new computer/devices.
Q32_12	Whether I am successful in the application of modern computers and devices depends essentially on me.

Participants answered using a 7-point Likert scale (Strongly Disagree, Disagree, Somewhat disagree, Neither agree nor disagree, Somewhat agree, Agree, and Strongly agree).

Technological readiness correlates with self-efficacy, one’s perception of their ability to effectively use technology. A comparable survey, administered in Germany, found significant levels of perceived usefulness. It has been used in several studies including^15^ and^16^. It is not clear that these results apply to populations with a lower socioeconomic status, significant racial minorities, or in the context of rural America.

The goal of including this scale in the survey is to understand how technology readiness may correlate with technology acceptance (through perceived usefulness and perceived ease of use).

#### Access and experience with technology and the Internet

Participants were also asked to self-report whether they had the following devices: computer, smartphone, internet, other computer devices. Responses were yes, no, or not sure. In addition, participants are asked about technology-related activities listed in Table 4.

**Table 4:** Questions on technology experiences and activities. Question 33 options were Yes, No, and Unsure.

*Technology ownership and access*	
Q33_1	Do you have the following devices? Computers
Q33_2	Do you have the following devices? Smart home
Q33_3	Do you have the following devices? Internet
Q33_4	Do you have the following devices? Other computer devices
*Have you participated in the following activities*..	
Q34_1	Used a step tracker
Q34_2	Read or posted on social media (Facebook, Twitter, etc)
Q34_3	Have a security system
Q34_4	Sent an email
Q34_5	Sent a text message
Q34_6	Searched for health information online
Q34_7	Made an appointment with doctor, government, etc
Q34_8	Purchased an item from the Internet (Amazon, Walmart, Easy, etc)

Question 34 answer options were Never, Seldom, Occasionally, Often, Recently, and Not Sure.

#### Big Five Personality Inventory

Personality traits were measured against the Big Five Personality Model. Participants answered questions on personality (Table 5) using the abbreviated 11-item Big Five Personality Inventory^5^, which is adapted from BFI-44 as a mechanism to measure the personality traits when time is a factor. This measures personality along 5 subscales: extraversion, openness, agreeableness, conscientiousness, and neuroticism. This instrument retains significant levels of reliability and validity against the original 44-item personality scale to verify that it.

**Table 5:** Personality questions used the abbreviated 11-item Big Five Inventory^5^.

Q35_1	I see myself as someone who: - ...is reserved
Q35_2	I see myself as someone who: - ...is generally trusting
Q35_3	I see myself as someone who: - ...tends to be lazy
Q35_4	I see myself as someone who: - ...is relaxed, handles stress well
Q35_5	I see myself as someone who: - ...has few artistic interests
Q35_6	I see myself as someone who: - ...is outgoing, sociable
Q35_7	I see myself as someone who: - ...tends to find fault with others
Q35_8	I see myself as someone who: - ...does a thorough job
Q35_9	I see myself as someone who: - ...gets nervous easily
Q35_10	I see myself as someone who: - ...has an active imagination
Q35_11	I see myself as someone who: - ...is considerate and kind to almost everyone

Each question is answered using a Likert scale (Disagree strongly, Disagree a little, Neither agree or disagree, Agree a little, or Agree strongly).

#### Sociodemographic characteristics

Survey participants self-reported their age, gender, and racial and ethnic origin. Rurality was determined by the site each participant was recruited from. Questions are in Table 6.

**Table 6:** Demographic questions regarding gender, age group, ethnicity, and education level.

Q36	What is your gender?	Female, Male
Q37	What is your age?	Under 18, 18-24, 25-34, 35-44, 45-54, 55-64, 65-74, 75-84, older than 85
Q38	What ethnicity to you primarily identify with?	White, Black or African American, American Indian or Alaska Native, Asian, Native Hawaiian or Pacific Islander, Other
Q39	What is your education level?	Less than high school, High school graduate, Some college, 2 year degree, 4 year degree, Professional degree, Doctorate

## Data Records

The dataset is published as an Excel spreadsheet in the Figshare repository^17^ (10.6084/m9.figshare.21365409). Rows 1 and 2 contain headings with question number and question text. Rows 5-122 contain the data. Fields where the participant did not provide an answer are blank.

Survey questions are listed in Tables 1–6.

The data is not coded so each field contains the text that corresponded to the answer selected. No analysis or manipulation of the data is present in the dataset.

## Technical Validation

We used validated and/or previously administered instruments where possible. Validation of the technological readiness and the BFI are detailed in^4^ and^5^. Smart device scenarios were reused from^3^ to provide a basis for comparison. Questions on smart device attitudes use the Technology Acceptance Model^12^ by asking questions on interest and usefulness. The remaining questions are demographic.

## Usage Notes

The spreadsheet enables preliminary exploration of the data in Excel using sort and filter. The spreadsheet can be imported into most software packages by exporting the spreadsheet into a CSV file(comma-separated values).

The data can be coded by replacing text with numerical values. This can be done in Excel. However, it may be more useful to import text data and code it in the analysis software. Data codes are better preserved when available in coding data and transformations are tracked.

Attitudes regarding smart devices and scenarios are collected using Likert-type scales. Parametric analytical techniques are appropriate for analyzing these responses. In addition, there is some evidence that groups of Likert-type responses can be treated as continuous data. Big Five Personality Inventory includes a scoring rubric that assigns a value to each of the five subscales. Due to copyright issues, consult^5^ Appendix A to link each item to one of the five traits and determine if a question is straight or reverse coded. The technological readiness instrument relates items 1-4 to the technology acceptance, items 5-8 to technology competence, and items 9-12 to technology control. Item numbers are identified in Table 3.

## Data Availability

No coding has been applied.
